# Parental Uveitis Influences Offspring With an Increased Susceptibility to the Experimental Autoimmune Uveitis

**DOI:** 10.3389/fimmu.2020.01053

**Published:** 2020-06-16

**Authors:** Guangnian Yin, Wenxin Zeng, Kaijiao Hu, Jie Gao, Jianping Liu, Yan Chen, Feilan Chen

**Affiliations:** ^1^Laboratory Animal Center, Chongqing Medical University, Chongqing, China; ^2^Chongqing Engineering Research Center for Rodent Laboratory Animals, Chongqing Medical University, Chongqing, China; ^3^Pathology Department, Chongqing Medical University, Chongqing, China; ^4^College of Pharmacy, Chongqing Medical and Pharmaceutical College, Chongqing, China

**Keywords:** experimental autoimmune uveitis (EAU), parental uveitis, offspring, differentially expressed gene (DEG), immune system, susceptibility

## Abstract

**Purpose:** Previous studies have shown that parental abnormal physiological conditions such as inflammation, stress, and obesity can be transferred to offspring. The purpose of this study was to investigate the impact of parental uveitis on the development and susceptibility to experimental autoimmune uveitis (EAU) in offspring.

**Methods:** Parental male and female B10RIII mice were immunized with interphotoreceptor retinoid binding protein (IRBP) 161–180 in complete Freund's adjuvant and were immediately allowed to mate. Gross examination of the offspring gestated with EAU was performed to determine the influence of parental uveitis on offspring development after birth. Gene expression profiles were analyzed in the affected eyes of offspring under EAU to identify differentially expressed genes (DEGs). Adult offspring were given 5, 25, and 50 μg IRBP_161−180_ to compare their susceptibility to EAU. Immunized mice were clinically and pathologically evaluated for the development of EAU. Ag-specific T-cell proliferation and IL-17 production from spleens and lymph nodes were evaluated on day 14 or 35 after immunization.

**Results:** Hair loss, delay of eye opening, and swollen spleens in the offspring from parents with uveitis were observed from day 14 to 39 after birth. DEGs were involved in the immune system process, muscle system process, and cell development. The altered antigen processing and presentation, cell adhesion molecules, and phagosome in the eyes of the offspring from uveitis-affected parents were enriched. Offspring gestated with EAU showed a susceptibility to EAU and an earlier onset and higher severity of EAU compared to the control group mice. IRBP-specific lymphocyte proliferation and IL-17 production were observed in the EAU offspring with exposure to parental uveitis.

**Conclusions:** The results suggest that mouse parents with uveitis can increase their offspring's susceptibility to EAU, probably through altering cell adhesion molecules and antigen processing and presentation related to the T-cell proliferation and Th17 response.

## Introduction

Non-infectious uveitis belongs to the class of sight-threatening autoimmune disorders; the condition may involve systemic syndromes or only eye symptoms that are influenced by interactions of genetic and environmental factors (1). Uveitis occurs frequently in adults of reproductive age and is the fourth most common cause of visual loss among the working-age population in the developed world (2). Previous studies have shown that paternal and or maternal metabolic syndrome, obesity, social stressors, anxiety, and inflammation can be passed on to offspring (3–6). There are also reports that the four main non-communicable diseases (cardiovascular diseases, cancers, diabetes, and chronic pulmonary disease) increase the potential risk for the generational transfer of aberrant maternal physiology (7, 8). Moreover, an earlier human study regarding inflammatory bowel disease (IBD) found that both parents having IBD had a higher impact on the susceptibility of children to the disease comparing to having only one parent or neither parent with IBD (9). However, it remains unclear whether parental uveitis affects the offspring later in life. Thus, the purpose of our study was to investigate the effect of parental uveitis on the development, gene expression profile, and susceptibility to uveitis in offspring later in life in a model of classical interphotoreceptor retinoid binding protein (IRBP)-induced parental experimental autoimmune uveitis (EAU). We observed hair loss, swollen spleens, and a delay of eye opening in the pups gestated with EAU. The differential gene expression of the eye in those offspring was most significantly enriched in the immune system process, muscle system process, and cell development. The increased susceptibility and Th17 cell immune response in EAU mice gestated with EAU were consistent with the upregulated genes related to antigen processing and presentation and the Th17 cell response.

## Materials and Methods

### Animals

B10.RIII mice (The Jackson Laboratory) were housed and maintained in specific pathogen-free conditions at Chongqing Medical University. We followed the guidelines of the National Institutes of Health and the ARVO Statement for the Use of Animals in Ophthalmic and Vision Research in regard to the animals' care and use. The Ethics Committee of Chongqing Medical University approved the protocol for the animal study (Permit number: 2015020).

### Induction of EAU in Mice

For induction of parental EAU, 8–12-week-old mice were subcutaneously immunized at the base of the tail and both thighs with 50 μg human IRBP_161−180_ (SGIPYIISYLHPGNTILHVD, Shanghai Sangon Biological Engineering Technology & Services Co., Ltd.) peptide in a 200 μL emulsion in complete Freund's adjuvant (CFA, St. Louis, MO, USA) containing 1.0 mg/mL mycobacterium tuberculosis (H37RA, ATCC 25177). The immunized female mice and male mice at a ratio of 3:2 were immediately kept together in cages for mating. The presence of vaginal plugs in mice was considered to indicate pregnancy, and that day was considered as pregnancy day 1. To investigate the impact of parental uveitis on the development of offspring, a gross examination of the offspring was performed every other day beginning from birth. To study the susceptibility to EAU in the offspring from parents suffering EAU, 7–8-week-old offspring gestated in the active EAU period (F1, days 7–20 post-immunization), offspring gestated during the remission of EAU (F2, equal to or more than 21 days after immunization), and offspring gestated from healthy control parents were subcutaneously immunized with 5, 25, and 50 μg human IRBP_161−180_ emulsified in 200 μL CFA containing 1.0 mg/mL mycobacterium tuberculosis.

### RNA Extraction

A total of three eyes from the F1 offspring gestated with EAU and the same number from healthy age-matched control offspring were collected to analyze the differential gene expression on day 29 after birth. The total RNA was extracted from the eyes using TRIzol^@^ reagent (Invitrogen, Carlsbad, CA, USA).

### Gene Expression Profile Analysis

RNA-sequencing (RNA-seq) was carried out by Origingene Biomedical Technology Co., Ltd. (Shanghai, China). The mRNA was enriched from the total RNA and reverse-transcribed into cDNA. Then, the cDNA was sequenced on an Illumina Hiseq X-Ten (LC Bio, China). Raw data in the FASTQ format were analyzed with a quality evaluation using FastQC v.0.11.4 and were filtered to obtain clean reads. The reads were mapped to the mouse genome GRCm38 in Ensemble92 using HISAT2 (10). Fragments per kilobase per million mapped reads (FPKM) values were utilized to calculate the expression of the genes in each sample. Genes with |log2 (fold change)| > 1 and a false discovery rate (FDR) < 0.05 were chosen as differentially expressed genes (DEGs) using the R package edgeR (11). The enrichment analysis of the Gene Ontology (GO) terms and pathways were performed by traditional singular enrichment analysis. Fisher's exact test was performed for the enrichment *P*-value calculation.

### Quantitative Polymerase Chain Reaction (q-PCR)

To validate the DEG library, the expression of eight DEGs and the endogenous control gene of GAPDH were performed by q-PCR in triplicate on an Applied Biosystems Bio-rad CFX 96 Real-Time PCR Detection System. The primers are listed in Supplementary Table 1. The 2^−ΔΔ*Ct*^ method was utilized to determine the relative gene expression.

### Clinical and Histological Examination

The immunized mice were checked two times per week to observe the clinical disease from day 7 after the immunization using a funduscope and a slit-light scope. Then immunized offspring were euthanized on day 14 post-immunization. Affected skin was collected for evaluation of histopathological changes on day 29 after birth. Eyeballs of the immunized offspring were harvested and fixed in 10% glutaraldehyde for 15 min and then immediately passed to 4% buffered formaldehyde for histopathology of the EAU. Paraffin-embedded skin and eye tissues were sliced into 4-μm sections for standard hematoxylin and eosin (H&E) staining. Scoring of clinical and histopathological grading in mice was determined in a masked manner on a scale from 0 to 4 following the previously reported criteria (12, 13).

### T Cell Proliferation

The spleens and lymph nodes from the EAU mice from both the F1 offspring group and the healthy control group were collected on day 14 after immunization. Antigen presenting cells (APCs) and T cells in the spleens and lymph nodes were enriched by passage through a nylon wool column, following the methods described in the literature (14). Next, 1 × 10^5^ APC cells were added to a 96-well-plate with final concentrations of 0, 10, and 20 μg/ml IRBP_161−180_, and then 4 × 10^5^ T cells were added to each well and incubated at 37°C in 5% CO_2_ for 72 h. The tetrazolium salt MTT was added, and the reaction was detected using an automatic microplate reader at a wavelength of 450 nm. The proliferation response was expressed as the mean proliferation stimulus index ± standard error of the mean (SEM) of the triplicate assays.

### Cytokine Production

The enriched APCs and T cells (as described above) at a ratio of 1:1 were added in the 6-well-plate with or without IRBP and cultured for 48 h. The cell supernatant was collected and detected for the IL-17 secretion using commercially available ELISA kits (R&D Systems, Minneapolis, MN).

### Statistical Analysis

The experiments were performed two or three times to validate the experimental data. Statistical analysis was conducted using SPSS version 17.0 for Windows (SPSS Inc., Chicago, IL, USA). Clinical and histopathologic scores were compared between the affected mice and control mice using a Mann–Whitney *U*-test or Kruskal-Wallis test. An unpaired Student's *t*-test for two groups or one-way analysis of variance (ANOVA) for three or more means were used for analysis. The level of statistical significance was set at *P* < 0.05.

## Results

### F1 Offspring Gestated With EAU Experienced Hair Loss and a Delay in Eye Opening

**Figure 1:**
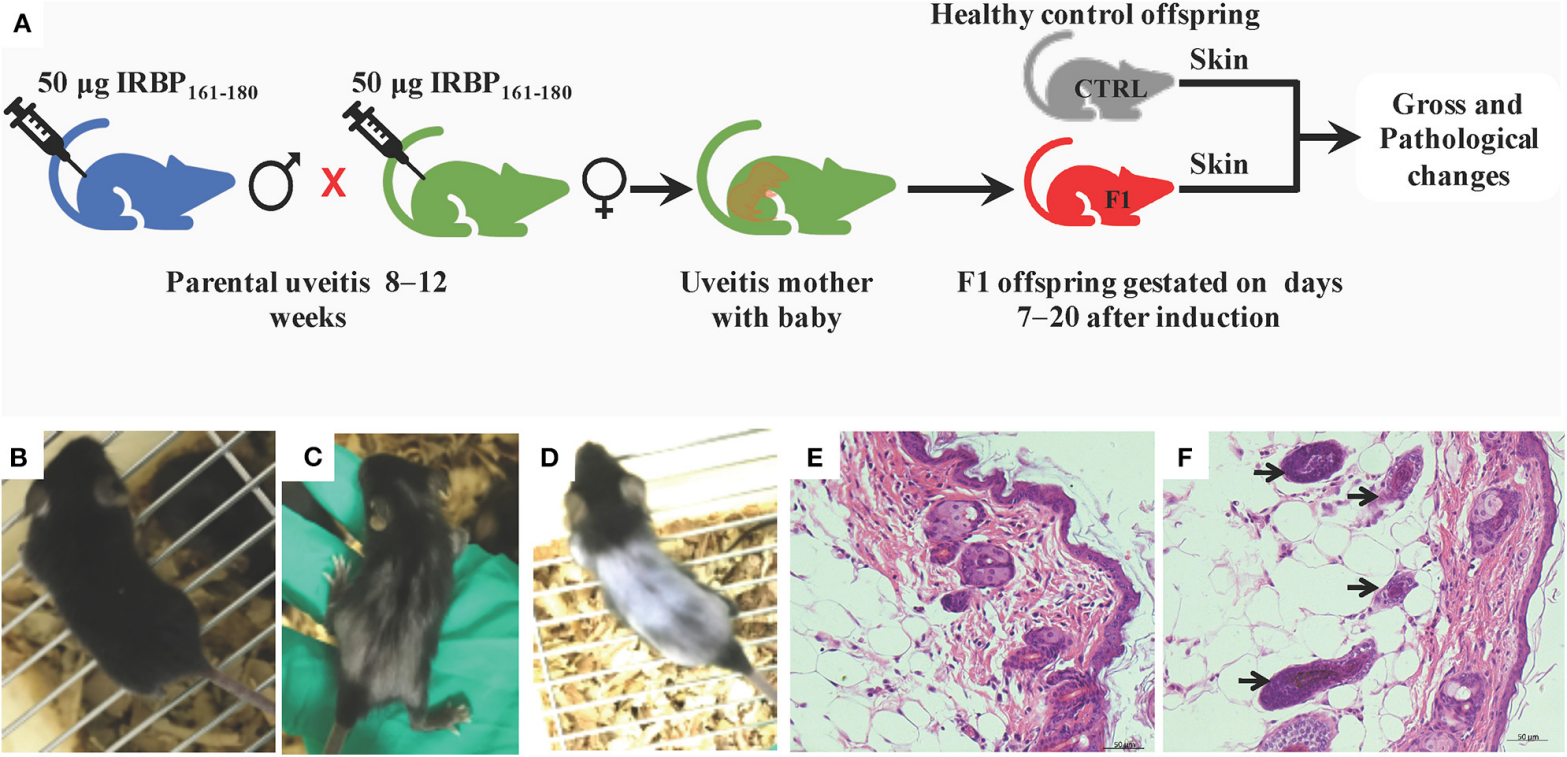
Hair appearance and histopathology (H&E) in B10R III mice. **(A)** Schematic illustration showing the workflow of gross and pathological examinations. **(B)** Normal hair in the healthy control group. **(C)** Hair loss in the F1 offspring gestated in parents with uveitis at day 17 and **(D)** day 35 after birth; **(E)** healthy skin at day 29; **(F)** the increased number of hair follicles in the subcutaneous tissue of F1 offspring at day 29 compared with the healthy control group (arrow).

To induce EAU in the parental mice, adult female and male mice were subcutaneously immunized with 50 μg IRBP and then allowed to mate (Figure 1A). The parental mice developed a classic EAU, with an onset on day 7 or 8, a peak on day 14, and a rapid resolution of active inflammation after immunization. The F1 offspring gestated during the active EAU period showed hair loss (Figures 1C,D) from days 14 to 39 and a delay of eye opening after birth compared with the healthy control mice (Figure 1B). Histopathology of the skin in regard to the hair loss in F1 offspring showed an increase in the number of hair follicles in the subcutaneous tissue (Figure 1F, arrow) compared with the skin of healthy age-matched control offspring (Figure 1E). A significantly swollen spleen was also observed in these F1 offspring. However, there were no significant abnormal presence in the gross appearance in the F2 offspring gestated during the remission of EAU.

### Enrichment of DEGs in F1 Offspring Gestated With EAU Occurred Mostly in the Immune System Process, Muscle System Process, and Cell Development

**Figure 2:**
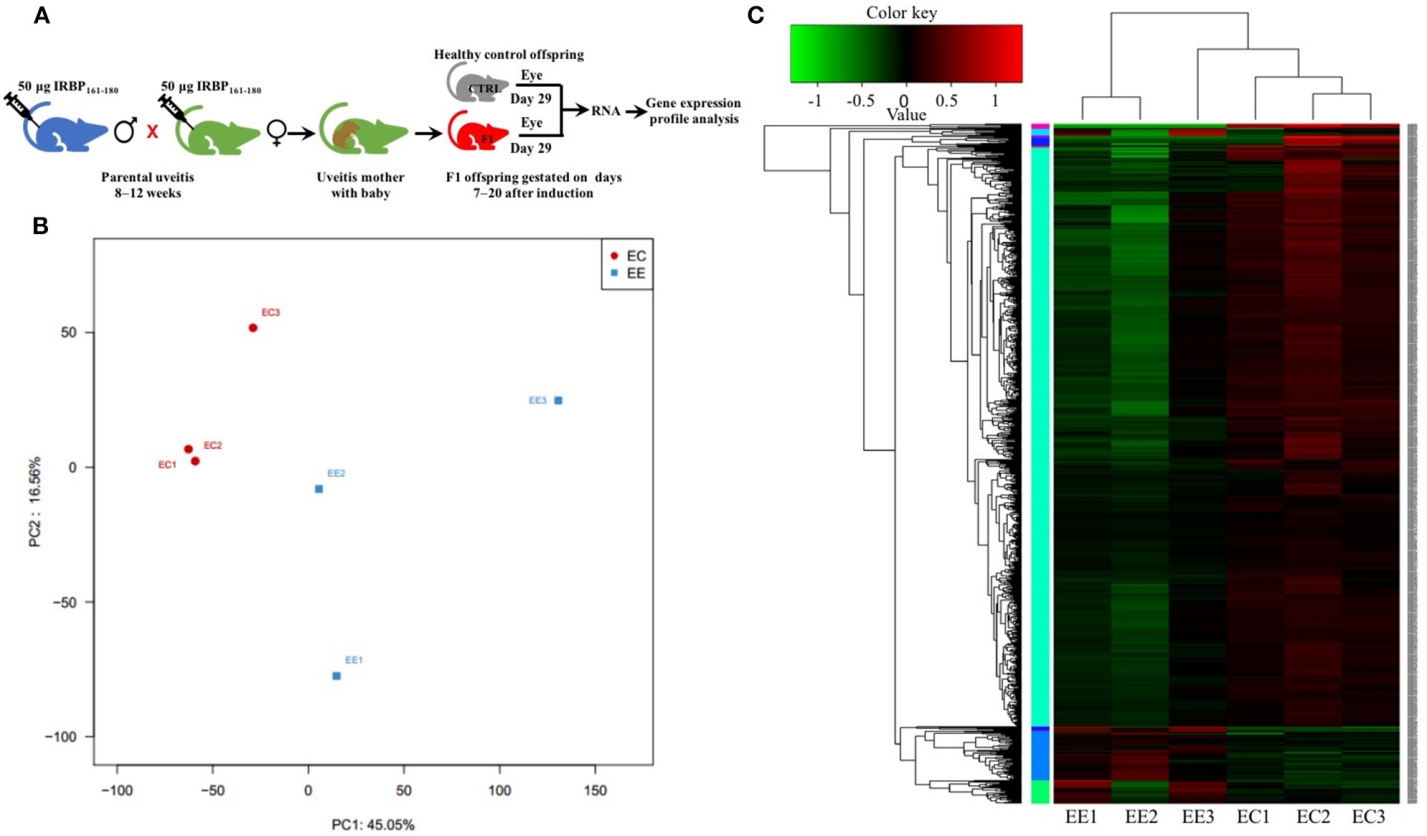
Principal component analysis (PCA) and heatmap of the differentially expressed genes (DEGs) in different samples. **(A)** Schematic illustration showing the workflow of RNA-seq of the eye. **(B)** PCA showing that the eye tissues from the same group clustered together. **(C)** The heatmap shows that the expression patterns were significantly different in the eyes of the affected offspring compared with those of the control. EE, Eyes in the experimental group. EC, Eyes in the healthy control group.

**Figure 3:**
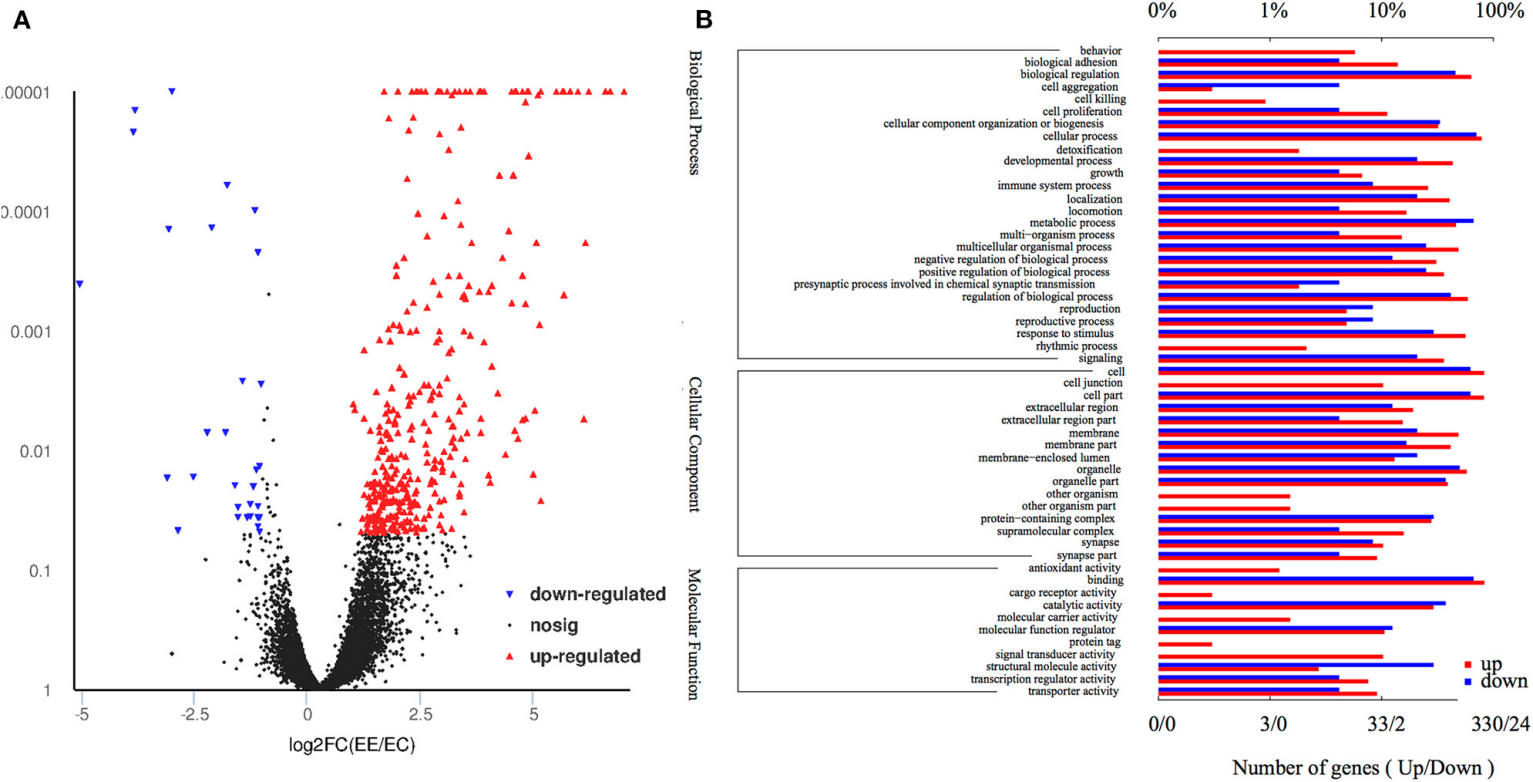
Volcano plot and gene ontology of the DEGs. **(A)** Volcano plot of the DEGs shows 363 upregulated genes (red dots) and 30 downregulated genes (blue dots) in the eyes of affected offspring compared with the control group. **(B)** Gene ontology of the 393 DEGs are involved in top 26 biological processes, 16 cellular components, and 11 molecular functions. The lower and upper abscissae show the numbers and ratios of annotated DEGs, respectively. The ordinate shows the categories of differential expression genes according to biological process, cellular components, and molecular function.

Differential gene expression analysis was performed using RNA-seq to understand how parental uveitis exposure affected offspring (Figure 2A). After the qualified control evaluation, the gene expression was obtained based on FPKM. The cluster of DEGs in the eye samples from the affected F1 offspring by parental EAU and control offspring from healthy parents showed that there was a high correlative index between the samples within the same group; however, there was a low correlative index between the samples between groups (Figure 2B). The heatmap revealed that the expression patterns were significantly different in the eyes of the affected offspring compared with those of the control (Figure 2C). A total of 393 DEG genes were identified (Figure 3A), and the top 20 DEGs in the eyes of the affected offspring compared to those of the control are presented in Table 1. The DEGs were shown to be chiefly associated with the GO terms “process,” “response,” “binding,” “development,” “complex,” “morphogenesis,” and “part” using the GO categories (Figure 3B). The enriched GO categories were spread across 586 biological processes, 111 cellular components, and 110 molecular functions; the top three enriched GO categories were the immune system process, muscle system process, and cytoskeletal protein binding (Table 2), which enabled us to further explore their potential role in EAU. As shown in Table 3, the DEGs in the immune system process were mostly related to the immune response, response to external stimuli, myeloid cell differentiation, antigen processing and presentation, T cell activation, and immune effector process. The transcript factor of Th17, the RAR-related orphan receptor alpha (RORα), was significantly increased in the offspring gestated with EAU.

**Table 1:** The top 20 differently expressed genes (DEGs) in eye from offspring gestated in parental uveitis.

**Gene symbol**	**Full name**	**FDR**	**log_**2**_FC**
Disp2	Dispatched RND tramsporter family member 2	2.04E-20	3.827775
Myh4	Myosin, heavy polypeptide 4, skeletal muscle	5.78E-16	6.218476
Tnnc2	Troponin C2, fast	3.63E-13	5.674978
Myl1	Myosin, light polypeptide 1	3.27E-11	4.758568
Actc1	Actin, alpha, cardiac muscle 1	1.05E-10	5.672237
Acta1	Actin, alpha 1, skeletal muscle	1.19E-10	6.009902
Myh2	Myosin, heavy polypeptide 2, skeletal muscle, adult	1.78E-09	6.611344
Mb	Myoglobin	2.33E-09	5.51296
H2-Ab1	Histocompatibility 2, class II antigen A, beta 1	8.42E-09	3.862867
Mylpf	Myosin regulatory light chain 2, skeletal muscle isoform	1.10E-08	4.642088
Tcap	Titin-cap	2.14E-08	5.51826
Gm3055	Predicted gene 3055	2.14E-08	3.09705
2810047C21Rik1	RIKEN cDNA 2810047C21 gene 1	2.35E-08	2.617525
Tnnt3	Troponin T3, skeletal, fast	2.35E-08	5.690388
Cox6a2	Cytochrome c oxidase subunit 6A2, mitochondrial	3.22E-08	4.887749
9430073C21Rik	Riken cDNA 9430073C21 gene	4.26E-08	3.365613
Atp2a1	ATPase, Ca^++^ transporting, cardiac muscle, fast twitch 1	4.73E-08	7.017263
Art1	ADP-ribosyltransferase 1	4.73E-08	3.497512
Rdh18-ps	Retinol dehydrogenase 18, pseudogene	4.73E-08	3.606664
Tcf4	Transcription factor 4	6.22E-08	2.30283

*FDR, false discovery rate; FC, fold change*.

**Table 2:** The top 7 function enrichment of different genes in eye from offspring gestated in parental uveitis.

**Description**	**FDR**	**No. of genes**	**Gene**
Immune system process	0	88	Abcc9, Alas2, Ank1, Apbb1ip, Apod, B2m, C1qa, C1qc, C1ra, Ccl11, Ccr2, Cd19, Cd274, Cd36, Cd74, Cfh, Ch25h, Col3a1, Creb1, Ctss, Cxcl12, Cxcl2, Dapk2, Epb42, Ets1, Flt1, Gbp3, Gbp4, Gbp7, Gbp9, Gm20547, Gm43302, Grap2, H2-Aa, H2-Ab1, H2-Q4, H2-Q6, H2-T24, Hba-a1, Hba-a2, Hrh2, Ifit1, Ifit1bl1, Iigp1, Il16, Inpp4b, Isg15, Isg20, Itgb2, Itgb3, Lcp1, Mb, Mpp1, Mylpf, Oasl2, Pagr1a, Parp14, Pf4, Pglyrp4, Ppbp, Ptprc, Rab3c, Rac2, Rbfox2, Rhag, Rora, Rps6ka3, Rsad2, Sfrp2, Six1, Snca, Sox6, Sp110, Spib, Stxbp1, Tgfbr2, Tgtp1, Tgtp2, Thbs4, Themis2, Tlr4, Tnf, Trim10, Trim30a, Trim58, Was, Wnt2b, Zfp36
Muscle system process	0	33	Acta1, Actc1, Atp2a1, Atp2b4, Calcrl, Col14a1, Csrp3, Hrc, Jsrp1, Kcna1, Kcnma1, Lmod2, Mb, Myh1, Myh2, Myh3, Myh4, Myh7, Myh8, Myl1, Mylk2, Myoc, Nos1, Ryr1, Scn4b, Smpx, Srl, Tcap, Tnnc2, Tnni2, Tnnt3, Trdn, Ttn
Cytoskeletal protein binding	0	50	Actc1, Ank1, Arl4c, Clmn, Csrp3, Des, Dnm3, Flnc, Hspb7, Kcnc1, Kcnma1, Kif5a, Lcp1, Ldb3, Lmod2, Mapt, Myh1, Myh13, Myh15, Myh2, Myh3, Myh4, Myh7, Myh8, Myo7b, Myoc, Myot, Myoz1, Myoz2, Neb, Nrap, Phactr1, Ptprt, Rab3c, Rcan3, Rhag, Slc4a1, Smtnl1, Snca, Synpo2l, Tcap, Tnnc2, Tnni2, Tnnt3, Tppp, Trim54, Ttn, Was, Wipf1, Xirp2
Actin cytoskeleton	0	35	Acta1, Actc1, Flnc, Flt1, Hspb7, Lcp1, Lmod2, Myadm, Myh1, Myh13, Myh15, Myh2, Myh3, Myh4, Myh7, Myh8, Myl1, Mylpf, Myo7b, Myoz1, Myoz2, Neb, Peak1, Rac2, Sept2, Snca, Srcin1, Synpo2l, Tnnc2, Tnni2, Tnnt3, Ttn, Was, Wipf1, Xirp2
Supramolecular complex	0	53	Abcc9, Acta1, Actc1, Ank1, Atp2a1, Atp2b4, Col3a1, Csrp3, Dcn, Des, Dnm3, Flnc, Hrc, Kif5a, Lcp1, Ldb3, Lmod2, Mapt, Myh1, Myh13, Myh2, Myh3, Myh4, Myh7, Myh8, Myl1, Myom3, Myot, Myoz1, Myoz2, Neb, Nos1, Nrap, Obscn, Rac2, Ryr1, Sept2, Slc4a1, Smpx, Smtnl1, Snca, Synpo2l, Tcap, Tnnc2, Tnni2, Tnnt3, Tppp, Trim54, Ttn, Tubb1, Was, Wipf1, Xirp2
Supramolecular polymer	0	53	Abcc9, Acta1, Actc1, Ank1, Atp2a1, Atp2b4, Col3a1, Csrp3, Dcn, Des, Dnm3, Flnc, Hrc, Kif5a, Lcp1, Ldb3, Lmod2, Mapt, Myh1, Myh13, Myh2, Myh3, Myh4, Myh7, Myh8, Myl1, Myom3, Myot, Myoz1, Myoz2, Neb, Nos1, Nrap, Obscn, Rac2, Ryr1, Sept2, Slc4a1, Smpx, Smtnl1, Snca, Synpo2l, Tcap, Tnnc2, Tnni2, Tnnt3, Tppp, Trim54, Ttn, Tubb1, Was, Wipf1, Xirp2
Supramolecular fiber	0	53	Abcc9, Acta1, Actc1, Ank1, Atp2a1, Atp2b4, Col3a1, Csrp3, Dcn, Des, Dnm3, Flnc, Hrc, Kif5a, Lcp1, Ldb3, Lmod2, Mapt, Myh1, Myh13, Myh2, Myh3, Myh4, Myh7, Myh8, Myl1, Myom3, Myot, Myoz1, Myoz2, Neb, Nos1, Nrap, Obscn, Rac2, Ryr1, Sept2, Slc4a1, Smpx, Smtnl1, Snca, Synpo2l, Tcap, Tnnc2, Tnni2, Tnnt3, Tppp, Trim54, Ttn, Tubb1, Was, Wipf1, Xirp2

*FDR, False discovery rate; No, Number*.

**Table 3:** Enrichment of different genes on multiple immune system process in eye from offspring gestated in parental uveitis.

**Description**	**FDR**	**No**.	**Genes**
Immune response	6.21E-09	54	Apbb1ip, B2m, C1qa, C1qc, C1ra, Ccl11, Ccr2, Cd19, Cd274, Cd36, Cd74, Cfh, Col3a1, Ctss, Cxcl12, Cxcl2, Gbp3, Gbp4, Gbp7, Gbp9, Gm20547, Gm43302, Grap2, H2-Aa, H2-Ab1, Hrh2, Ifit1, Iigp1, Isg15, Isg20, Lcp1, Mylpf, Oasl2, Pagr1a, Parp14, Pf4, Pglyrp4, Ppbp, Ptprc, Rac2, Rora, Rps6ka3, Rsad2, Snca, Sp110, Stxbp1, Tgtp1, Tgtp2, Themis2, Tlr4, Tnf, Trim10, Trim30a, Was
Response to external stimulus	1.74E-08	74	Abcc9, Acta1, Apod, B2m, Calcrl, Ccl11, Ccr2, Cd36, Cd74, Cfh, Ch25h, Cmpk2, Cntn2, Col3a1, Creb1, Csrp3, Ctss, Cxcl12, Cxcl2, Dapk2, Dcc, Dclk1, Dcn, Epha5, Ets1, Flt1, Gbp3, Gbp4, Gbp7, Gbp9, Gm43302, Ifi27l2a, Ifit1, Ifit1bl1, Iigp1, Il16, Isg15, Isg20, Itgb3, Kcna1, Kcnc1, Kif5a, Lyz2, Mpp1, Myh13, Nrxn1, Oasl2, Pf4, Pglyrp4, Postn, Ppbp, Rac2, Rora, Rps6ka3, Rsad2, Scd1, Sfrp2, Snca, Sp110, Tbr1, Tcap, Tgfbr2, Tgtp1, Tgtp2, Thbs4, Tlr4, Tnf, Tnr, Trim30a, Tspan8, Ttn, Wipf1, Wnt2b, Zfp36
Defense response	9.09E-08	54	ts1, Gbp3, Gbp4, Gbp7, Gbp9, Gm43302, Grap2, H2-Aa, H2-Ab1, Ifi47, Ifit1, Ifit1bl1, Iigp1, Isg15, Isg20, Itgb2, Lyz2, Oasl2, Parp14, Pf4, Pglyrp4, Ppbp, Rora, Rps6ka3, Rsad2, Scd1, Snca, Sp110, Stxbp1, Tgtp1, Tgtp2, Themis2, Tlr4, Tnf, Trim10, Trim30a, Was, Zfp36
Regulation of immune system process	4.63E-07	47	Apod, B2m, C1qa, C1qc, C1ra, Ccr2, Cd19, Cd274, Cd36, Cd74, Cfh, Col3a1, Creb1, Cxcl12, Cxcl2, Dapk2, Ets1, Gbp4, Gm20547, H2-Aa, H2-Ab1, Inpp4b, Isg15, Itgb3, Mpp1, Pagr1a, Parp14, Pf4, Pglyrp4, Ppbp, Ptprc, Rac2, Rbfox2, Rora, Rps6ka3, Rsad2, Snca, Stxbp1, Tgfbr2, Thbs4, Themis2, Tlr4, Tnf, Trim30a, Trim58, Was, Zfp36
Response to cytokine	1.31E-06	39	Aa, H2-Ab1, Ifi209, Ifi47, Ifit1, Iigp1, Isg15, Laptm5, Parp14, Pf4, Postn, Ppbp, Rora, Snca, Stxbp1, Tgtp1, Tgtp2, Tnf, Was, Zfp36
Innate immune response	1.52E-06	33	C1qa, C1qc, C1ra, Ccl11, Cd36, Cd74, Cfh, Gbp3, Gbp4, Gbp7, Gbp9, Gm43302, Grap2, H2-Aa, H2-Ab1, Ifit1, Iigp1, Isg15, Isg20, Oasl2, Parp14, Pglyrp4, Rps6ka3, Rsad2, Snca, Sp110, Stxbp1, Tgtp1, Tgtp2, Tlr4, Trim10, Trim30a, Was
Positive regulation of immune system process	1.95E-06	36	B2m, C1qa, C1qc, C1ra, Ccr2, Cd19, Cd274, Cd36, Cd74, Cfh, Creb1, Cxcl12, Cxcl2, Dapk2, Ets1, Gm20547, H2-Aa, H2-Ab1, Isg15, Itgb3, Pagr1a, Pf4, Pglyrp4, Ppbp, Ptprc, Rac2, Rps6ka3, Rsad2, Stxbp1, Tgfbr2, Thbs4, Themis2, Tlr4, Tnf, Trim30a, Trim58
Response to interferon-gamma	7.53E-06	14	Ccl11, Gbp3, Gbp4, Gbp7, Gbp9, Gm43302, H2-Aa, H2-Ab1, Parp14, Snca, Stxbp1, Tgtp1, Tgtp2, Was
Regulation of response to stimulus	1.66E-05	93	ora, Rps6ka3, Rsad2, Sept2, Sfrp2, Snca, Sp100, Srl, Stxbp1, Syt4, Tbr1, Tcf4, Tgfbr2, Thbs4, Themis2, Tlr4, Tnf, Tnr, Trdn, Trim30a, Tspan8, Was, Wnt2b, Wnt7a, Xdh, Zfp36
Response to stimulus	1.90E-05	193	bt, Hfe2, Hrc, Hrh2, Htr1b, Ifi209, Ifi27l2a, Ifi47, Ifit1, Ifit1bl1, Iigp1, Il16, Inpp4b, Isg15, Isg20, Itgam, Itgb2, Itgb3, Jsrp1, Kcna1, Kcnc1, Kcnd2, Kcnma1, Kdr, Kif5a, Laptm5, Lcp1, Lyz2, Mapt, Mb, Mmrn1, Mpp1, Myadm, Myh13, Myh2, Myh4, Myh7, Mylk2, Mylpf, Myoc, Ndrg4, Nos1, Nrxn1, Oasl2, Obscn, Pagr1a, Pak3, Parp14, Pde11a, Pf4, Pglyrp4, Pgr, Postn, Pou3f2, Ppbp, Ppp1r15a, Ptprc, Ptprn2, Ptprt, Rac2, Rasgrp3, Rbfox2, Rcan3, Retnla, Rhag, Rora, Rps6ka3, Rsad2, Ryr1, Scd1, Sept2, Sfrp2, Six1, Slc1a1, Slc6a14, Slfn14, Smtnl1, Snca, Sox6, Sp100, Sp110, Sparcl1, Srl, Stxbp1, Syt4, Tbr1, Tcap, Tcf4, Tgfbr2, Tgtp1, Tgtp2, Thbs4, Themis2, Tlr4, Tnf, Tnmd, Tnr, Trdn, Trim10, Trim30a, Tspan8, Ttn, Was, Wipf1, Wnt2b, Wnt7a, Xdh,Zfp36
Immune effector process	2.44E-05	29	Gm20547, H2-Ab1, Ifit1, Ifit1bl1, Isg15, Isg20, Lcp1, Oasl2, Pagr1a, Pglyrp4, Rac2, Rora, Rsad2, Stxbp1, Tlr4, Tnf, Trim30a, Was
Cellular response to cytokine stimulus	5.30E-05	32	Ab1, Ifi209, Ifi47, Ifit1, Iigp1, Laptm5, Parp14, Pf4, Postn, Ppbp, Rora, Stxbp1, Tgtp1, Tgtp2, Tnf, Was, Zfp36
MHC protein complex	5.40E-05	6	B2m, Cd74, H2-Aa, H2-Ab1, H2-Q4, H2-Q6
Regulation of response to external stimulus	7.40E-05	28	Apod, Calcrl, Ccr2, Cd36, Cd74, Cfh, Ctss, Cxcl12, Cxcl2, Dapk2, Ets1, Gbp4, Il16, Mpp1, Nrxn1, Pf4, Ppbp, Rac2, Rora, Snca, Tbr1, Tgfbr2, Thbs4, Tlr4, Tnf, Tnr, Tspan8, Zfp36
Antigen processing and presentation	0.0001	10	B2m, Cd74, Ctss, H2-Aa, H2-Ab1, H2-Q4, H2-Q6, H2-T24, Rab3c, Was
Intracellular region of host	0.000213	5	Gbp3, Gbp7, Gbp9, Iigp1, Pf4
Regulation of leukocyte adhesion to vascular endothelial cell	0.004	4	Ccr2, Cxcl12, Ets1, Tnf
MHC class II protein complex	0.007	3	Cd74, H2-Aa, H2-Ab1
MHC class I protein complex	0.0091	3	B2m, H2-Q4, H2-Q6
Regulation of NLRP3 inflammasome complex assembly	0.00914	3	Cd36, Tlr4, Trim30a
Antigen processing and presentation of peptide or polysaccharide antigen via MHC class II	0.028	3	Cd74, H2-Aa, H2-Ab1
Regulation of leukocyte tethering or rolling	0.042	2	Ccr2, Cxcl12

*FDR, False discovery rate; No, Number*.

**Figure 4:**
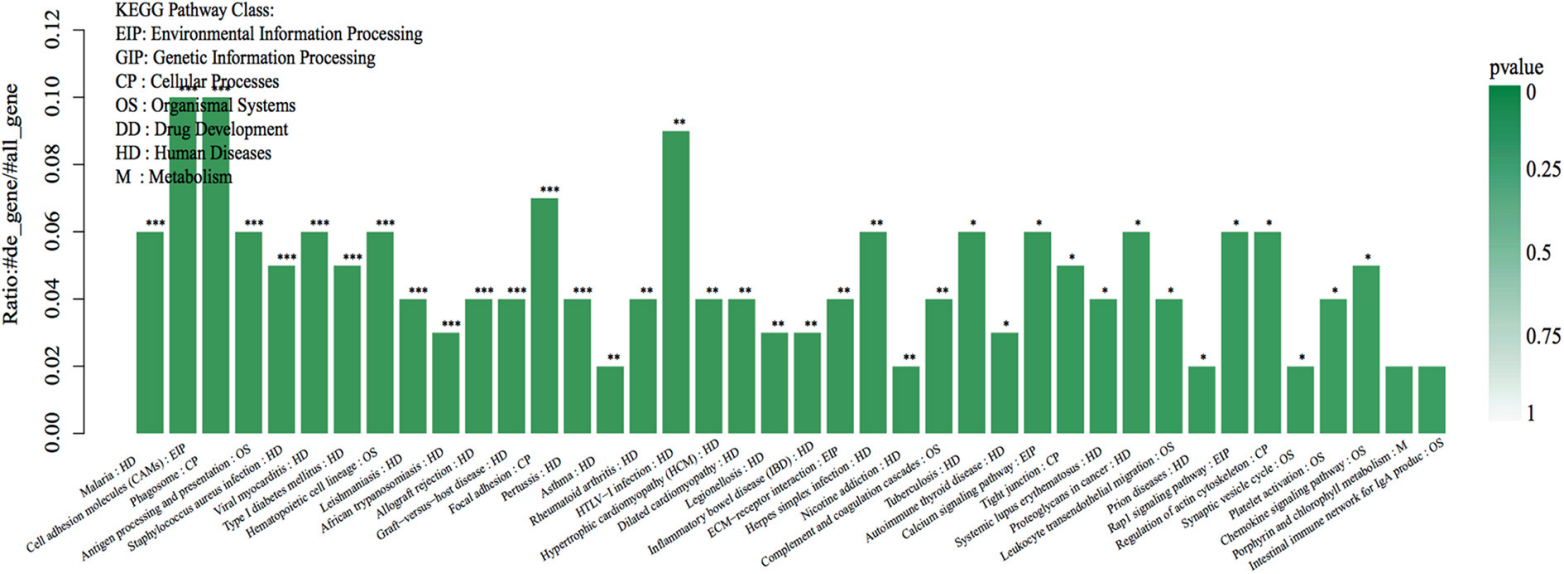
The top 40 KEGG pathway enrichments of the DEGs. The DEGs were enriched in 23 human diseases, 4 environmental information processes, 4 cellular processes, and 7 organismal systems by enrichment analysis of GO pathways. The abscissa represents the name and class of the KEGG pathway enrichment of the DEGs. The ordinate represents the enrichment of the ratio calculated by the formula of enrichment ratio = sample number/background number). ***False discovery rate (FDR) < 0.001, **FDR < 0.01, and *FDR < 0.05 from R package edge R.

As shown in the KEGG enrichment pathway of the mRNAs (Figure 4), 14 human diseases, including diabetes mellitus, cell adhesion molecules in environmental information processing, phagosome and focal adhesion in cellular processes, antigen processing and presentation, and hematopoietic cell lineage in organismal systems were significantly enriched in the affected eye group.

### Verification of DEGs by q-PCR

**Figure 5:**
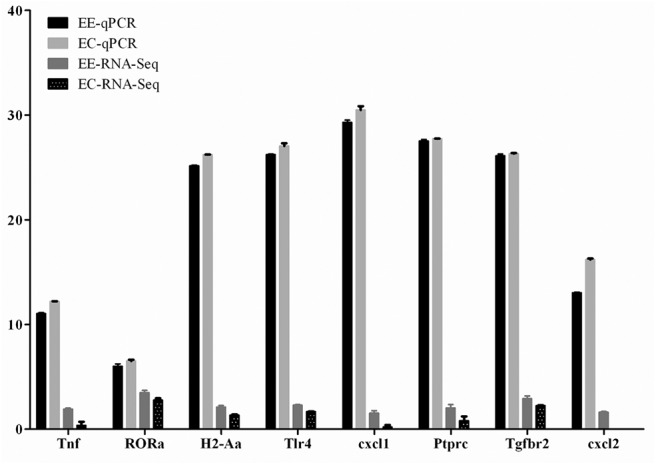
Validation of the differentially expressed genes by q-PCR. Expression levels of eight mRNAs were measured by q-PCR, named EE-qPCR and EC-qPCR. The mRNA levels were expressed as the means ± SEM of Δ*Ct* in three biological replicates. The clean reads of each mRNA molecule from RNA-Seq were also expressed as the means ± SEM on a log10 scale, named EE-RNA-seq and EC-RNA-seq.

The expression levels of eight DEGs were detected by q-PCR to determine the content of the DEG library. Figure 5 shows the same expression patterns in the q-PCR and RNA-seq analyses, thereby validating our results.

### Increased Susceptibility and Severity of EAU in Offspring Gestated With EAU

**Figure 6:**
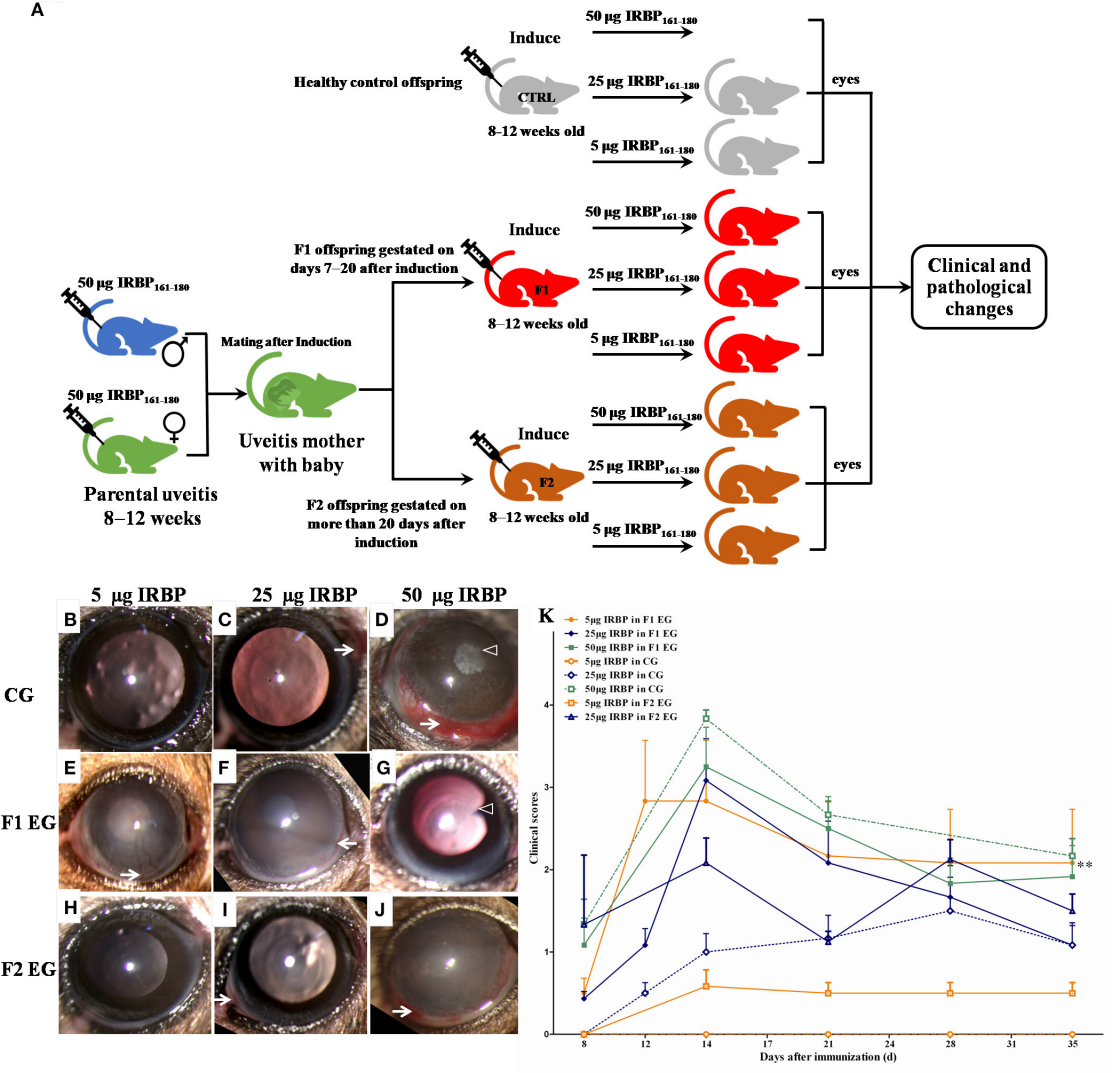
Clinical signs and clinical scores in the healthy control and affected offspring after immunization with different doses of IRBP. **(A)** Schematic illustration of the experimental design. **(B–J)** Representative anterior segment pictures of the control offspring (**B–D**: 5, 25, and 50 μg IRBP) and the affected the F1 offspring (**E–G**: 5, 25, and 50 μg IRBP) and the F2 offspring (**H–J**: 5, 25, and 50 μg IRBP) made with a slit lamp on day 14 after immunization. Arrows show vasculitis. Triangles show posterior synechiae. **(K)** Clinical scores were determined using a funduscope from day 7 to 35 after immunization. Data are shown as mean ± SEM (*n* = 6) and are representative of two independent experiments. **P < 0.01 in Kruskal-Wallis test.

**Figure 7:**
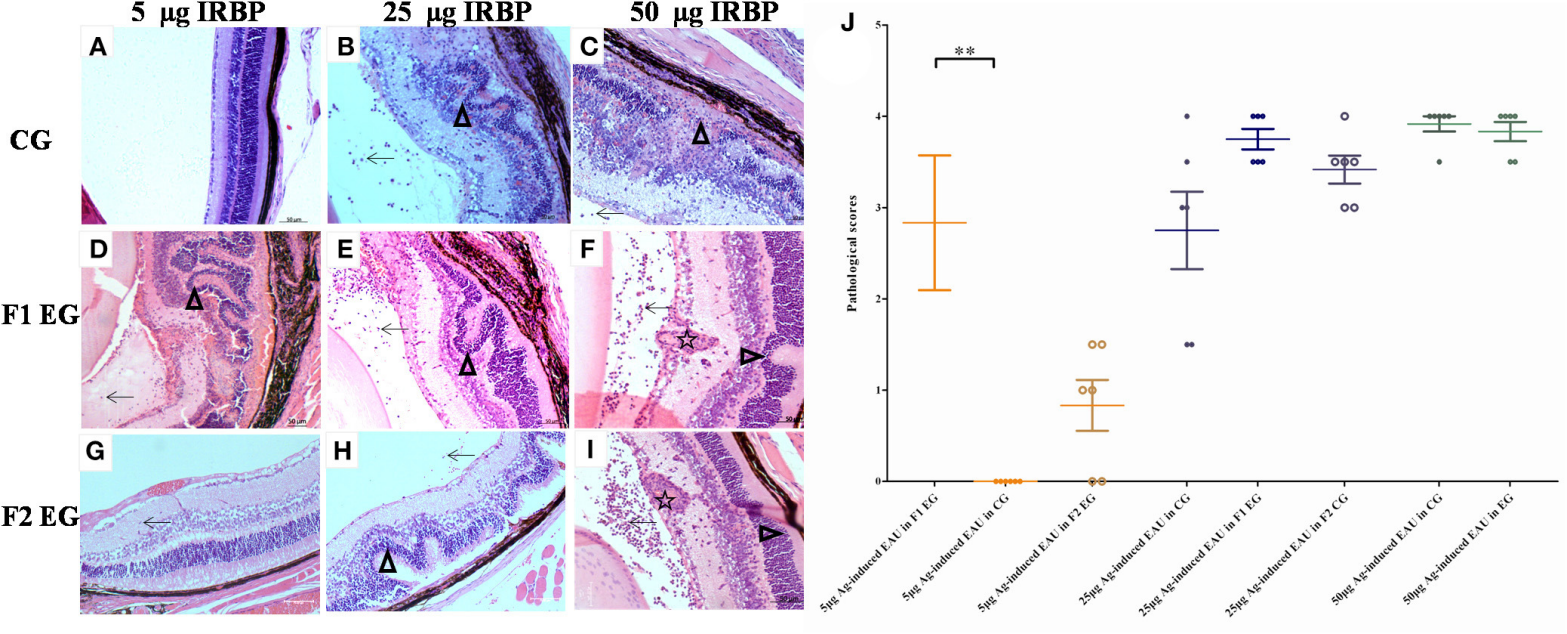
Histological pictures in the control and affected mice on day 14 following immunization. Histological images of the control groups (**A–C**: immunized with 5, 25, and 50 μg IRBP, respectively) and affected F1 mice (**D–F**: immunized with 5, 25, and 50 μg IRBP, respectively) and F2 mice (**G–I**: immunized with 5, 25, and 50 μg IRBP, respectively). **(J)** Histological scores were assessed on sections stained with hematoxylin and eosin from the control and experimental mice. Arrows show an infiltration of inflammatory cells. The star shows a granulomatous lesion. Triangles show the retinal folding or damage. Data are shown as the mean ± SEM (*n* = 6) and are representative of two independent experiments. **P < 0.01 in Kruskal-Wallis test.

To evaluate the effect of parental uveitis on the susceptibility of offspring to EAU, we immunized the F1 and F2 offspring gestated in the active and inactive EAU periods and healthy control offspring gestated in healthy parents with 5, 25, and 50 μg IRBP in CFA emulsification (Figure 6A). As shown in Figures 6, 7, both the control (Figures 6D, 7C) and the affected groups (Figures 6G,J, 7F,I, F1 and F2, respectively) induced with 50 μg IRBP presented a similar pattern both for the occurrence and the intensity of EAU symptoms (Figure 6K). In contrast, the F1 and F2 offspring gestated in the parental EAU period developed EAU disease with a severe clinical symptoms (Figures 6E,F, 5 and 25 μg in F1 offspring, respectively; Figures 6H,I, 5 and 25 μg in F2 offspring, respectively) and pathological grade (Figures 7D,E, 5 and 25 μg in F1 offspring, respectively; Figures 7G,H, 5 and 25 μg in F2 offspring, respectively) compared with heathy offspring immunized with 5 or 25 μg IRBP (Figures 6B,C, clinical grade; Figures 7A,B, pathological grade). However, the control offspring immunized with 5 μg IRBP did not develop an autoimmune uveitis (Figures 6B,K, 7A,J). In addition, EAU in the affected F1 and F2 offspring immunized with 25 μg IRBP showed an earlier onset of uveitis (on day 8) compared to the control group (Figure 6K).

### Offspring Gestated With EAU Show IRBP-Specific T Cell Proliferation and Th17 Response After EAU Induction

**Figure 8:**
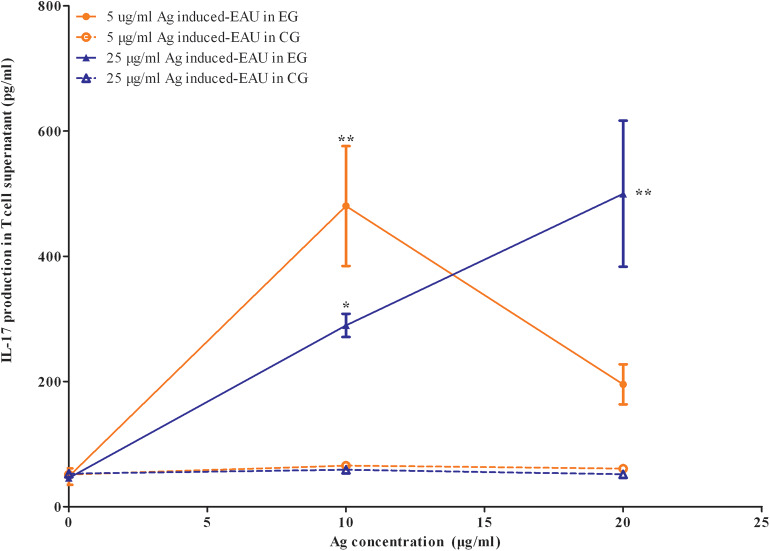
Offspring gestated with EAU produced IL-17 stimulated by the systemic IRBP-specific immune response *in vitro*. Lymphocytes from EAU-affected mice induced with 5 μg IRBP and 25 μg IRBP were collected on day 35 post-immunization and were stimulated with or without the IRBP peptide. IL-17 production was measured with an ELISA assay. Affected offspring indicated IL-17 production. Data are the mean ± SEM and are representative of two independent experiments. *P < 0.05, **P < 0.01 in one-way ANOVA. Three mice were used in each group.

**Figure 9:**
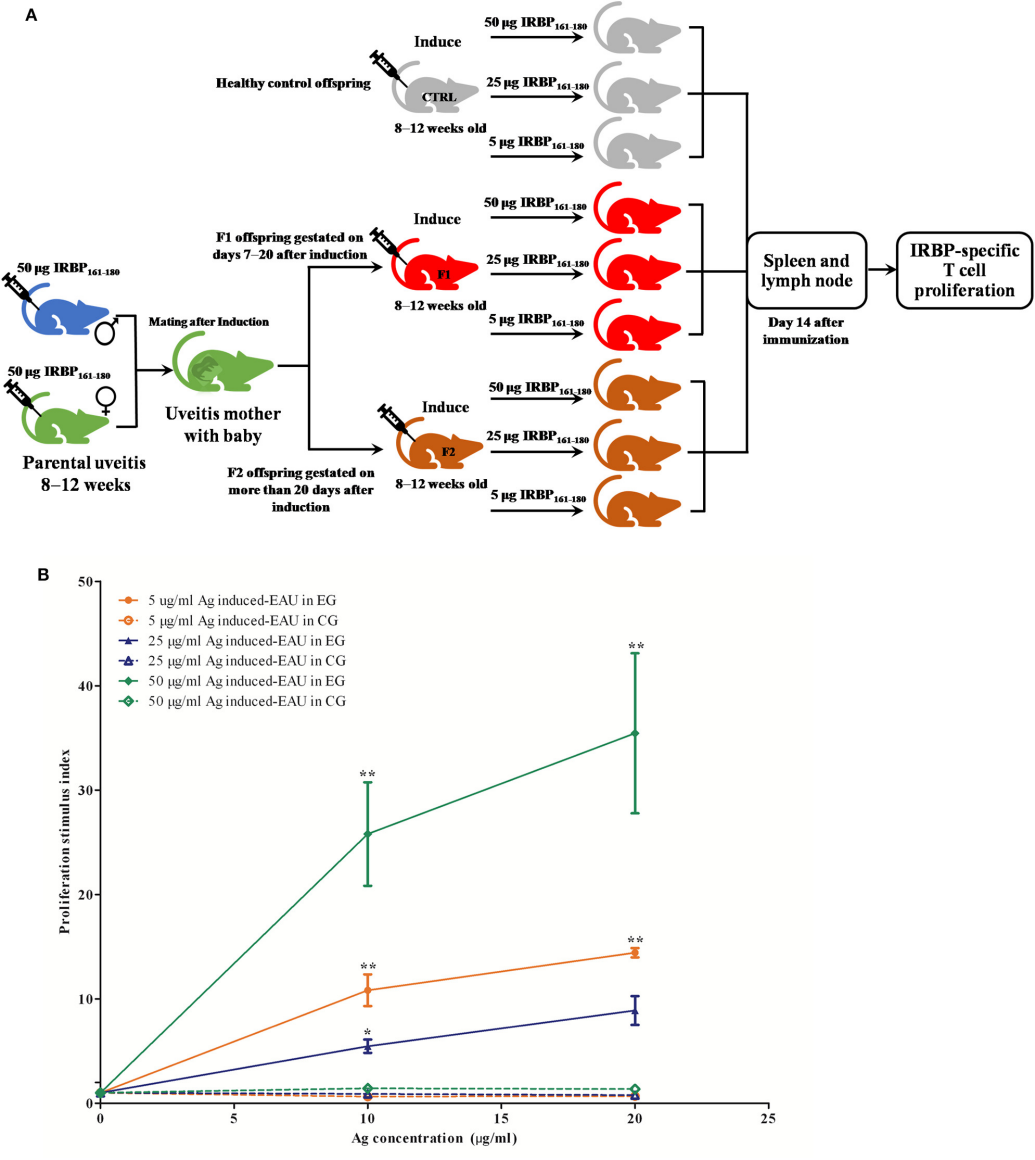
Offspring gestated with EAU showed a systemic IRBP-specific immune response *in vitro*. **(A)** Schematic illustration of the experimental design. **(B)** Lymphocytes from EAU-affected mice induced with 5 μg IRBP, 25 μg IRBP, and 50 μg IRBP were collected on day 14 post-immunization and were stimulated with or without the IRBP peptide. The proliferative response was measured via the MTT assay. The affected offspring showed the IRBP-specific proliferative response. Data are expressed as mean ± SEM and are representative of two independent experiments. *P < 0.05, **P < 0.01 in one-way ANOVA. Three mice were used in each group.

The APCs and T cells of mice gestated in the EAU and gestated in healthy parents were collected on day 14 or day 35 after immunization with 5, 25, and 50 μg IRBP and were *in vitro* co-cultured with or without IRBP. IL-17 was measured with ELISA kits in a supernatant of APC-T-cell co-culture system for 48 h. We found an increased production of IL-17 by the T cells from F1 offspring in 5-μg-Ag-induced and 25-μg-Ag-induced EAU in the presence of 10 μg IRBP_161−180_ and in 25-μg-Ag-induced EAU in the presence of 20 μg IRBP_161−180_ compared to the healthy control offspring, respectively (Figure 8, *P* < 0.05, one-way ANOVA). The T-cell proliferation assay was determined by MTT stimulated by APC with or without IRBP for 72 h. We found significantly higher proliferation of T-cells in the F1 offspring EAU group immunized with 5, 25, and 50 μg IRBP in the presence of 10 μg IRBP and immunized with 5 and 50 μg IRBP in the presence of 20 μg IRBP compared with the healthy control offspring, respectively (Figure 9, *P* < 0.05, one-way ANOVA).

## Discussion

We investigated the influence of parental uveitis on the development and susceptibility of offspring to EAU in B10RIII mice. The results indicated that parental uveitis caused hair loss, delay of eye opening, and aberrant gene expression that was mostly enriched in the immune system process, muscle system process, and cell development in the eyes of the affected offspring. Moreover, the affected offspring showed a susceptibility to EAU when induced by a lower dose (5 μg IRBP_161−180_) compared to the control offspring.

Non-infectious uveitis encompasses specific ocular syndromes and uveitis associated with systemic autoimmune diseases, such as Bechet's disease, ankylosing spondylitis (AS), psoriasis, inflammatory bowel disease (IBD), juvenile idiopathic arthritis (JIA), sarcoidosis, systemic lupus erythematosus, and tubulointerstitial nephritis and uveitis (TINU). An earlier epidemiological study on AS with associated diseases, including IBD, psoriasis, and iritis revealed that women with AS have a higher impact on the susceptibility of children to this disease than do men (15). Male patients with AS have a greater impact on the susceptibility of children to psoriasis. Iritis in children can be equally inherited from mothers and fathers with AS. Previous familial aggregation studies reported that children can inherit uveitis from their mothers with JIA-uveitis or TINU (16, 17). However, another human study demonstrated that both parents having IBD conferred a higher risk for the development of the disease in children compared to having only one parent or neither parent with IBD (9). Therefore, we first evaluated the influence of both parents with EAU on the susceptibility of offspring to EAU. However, which of the two parents is more important or whether they are equally important for offspring to develop EAU needs to be explored in future study.

Human and animal research has indicated that maternal stress during pregnancy alters the brain morphology, structure, and behavior of offspring in later life (18, 19). Similar to the results of previously reported studies, hair loss and the postponement of eye opening in offspring exposed to parental uveitis during gestation were observed on days 14–39 after birth in the present study. The upregulated genes, such as homeobox protein aristaless-like 4 related to hair follicle development (20), follistatin l5 (21), and Wnt transcription factor 4 related to hair follicle differentiation (22, 23) in the DEGs may provide the evidence for hair loss in F1 offspring gestated with parental uveitis. In addition, the functions of different gene expression profiles in the eye were also enriched in regard to the muscle system processes related to muscle contraction, muscle structure development, skeletal muscle contraction, and muscle filament sliding. Of those DEGs, the Col14a1 gene encoding the Collagen alpha-1 (XIV) chain and the Col3a1 gene encoding the Collagen alpha-1(III) chain were upregulated in the eyes of offspring gestated with EAU compared with the controls. Our results are consistent with the previous reports that the main constituents of the tendons and collagen types I, IV, VI, and XII were increased in the strabismic extraocular muscles (24–26). Therefore, we propose that those upregulated genes may cause the abnormal function of the extraocular muscles, and thus they may account for the postponement of eye opening in the offspring.

The functions of DEGs in the eyes of offspring mice were mostly enriched in the immune system process, which included the immune response, dendritic cell differentiation, innate immune response, positive regulation of the immune system process, response to interferon-gamma, immune effector process, and MHC protein complex. Those enriched functions in the affected eye are supported by the results of function enrichment of DEGs in the swollen spleens that contained immune system process, immune response, innate immune response, response to external stimulus, regulation of multicellular organismal process, and developmental process, suggesting that the shared enriched immune system process may be involved in the susceptibility to EAU in offspring (data unpublished). In particular, the genes CD74, H2-Aa, and H2-Ab1 are known to be involved in antigen processing and the presentation of peptides or polysaccharide antigens, suggesting that these genes may increase the response to external stimuli. Therefore, we hypothesized that the increased expression of those genes might increase the susceptibility to EAU in the offspring affected by parental uveitis. To validate this hypothesis, B10R III mice were immunized with 5, 25, and 50 μg IRBP_161−180_. The affected offspring gestated with EAU, but not the control offspring, developed EAU after immunization with 5 μg IRBP peptide, suggesting that these upregulated genes in the affected offspring with parents suffering from uveitis may cause the susceptibility to EAU. Our results are also in accordance with a study that showed gestational hypothyroxinemia not only increases the severity of EAE but also contributes to earlier-onset and more intense EAE (27). In addition, the report that chronic gestational inflammation can be transferred to the offspring also supports our results (6). The findings in our study and the previous reports support the notion that the offspring gestated with EAU shared common characteristics of multiple chronic diseases resulting from imprinting from the parental condition.

In addition, in alignment with the significantly-enriched CAMs and the antigen processing and presentation involved in T-cell receptor signaling-dependent proliferation, the increased IRBP-specific T-cell proliferation in the offspring gestated with EAU was also observed in the present EAU model. Finally, the upregulated gene Rorα regulating the Th17 cell differentiation and immune response may provide the evidence of the increased levels of the cytokine of IL-17 in the supernatant from enriched T cells in the offspring influenced by parental uveitis. However, the underlying mechanism of how those DEGs increase the susceptibility of their offspring to this disease needs to be explored in the future studies.

In conclusion, the results of the current study indicate that parental uveitis may increase the susceptibility of the offspring to EAU through multiple functions of DEGs enriched through the immune system process, particularly via T-cell proliferation and IL-17 production. Our findings provide evidence for the transgenerational impact on offspring of parents with uveitis, and identified possible risk factors for the prediction of a uveitis phenotype in future generations.

## Data Availability Statement

The datasets generated for this study can be found in the NCBI, BioSample accessions: SAMN13874768, SAMN13874769, SAMN13874770, SAMN13874771, SAMN13874772, SAMN13874773, https://www.ncbi.nlm.nih.gov/biosample/13874768, https://www.ncbi.nlm.nih.gov/biosample/13874769, https://www.ncbi.nlm.nih.gov/biosample/13874770, https://www.ncbi.nlm.nih.gov/biosample/13874771, https://www.ncbi.nlm.nih.gov/biosample/13874772, https://www.ncbi.nlm.nih.gov/biosample/13874773.

## Ethics Statement

The animal study was reviewed and approved by The Ethics Committee of Chongqing Medical University.

## Author Contributions

FC designed and performed the experiments, performed the data analysis, and wrote the manuscript. GY performed the experiments, was involved in data analysis, and wrote the manuscript. WZ, JG, KH, JL, and YC participated in the experiments. All authors have given permission to be named.

## Conflict of Interest

The authors declare that the research was conducted in the absence of any commercial or financial relationships that could be construed as a potential conflict of interest.
