# Supplementary material for: Parental Uveitis Influences Offspring With an Increased Susceptibility to the Experimental Autoimmune Uveitis
Source: Front Immunol. 2020 Jun 16;11:1053. doi: 10.3389/fimmu.2020.01053 (PMC7308830; doi:10.3389/fimmu.2020.01053)
Supplement: Supplementary file 1 [file Table_1.doc]

**Supplementary Table 1. Primers used for Verification of DEGs by q-PCR**

| Gene | sequence | PCR Products (bp) |
| --- | --- | --- |
| TNF-α (Tnf) | Forward sequence (5'-3'): AGCCGATGGGTTGTACCTTG  Reverse sequence (5'-3'): ATAGCAAATCGGCTGACGGT | 99 |
| MHC-II (H2-Aa) | Forward sequence (5'-3'): TGGCTCAGAAATAGCAAGTCAG  Reverse sequence (5'-3'): TAAATGTCATCGTCAGAAGGGA | 122 |
| Tlr4 | Forward sequence (5'-3'): CGCTCTGGCATCATCTTCA  Reverse sequence (5'-3'): TTTTCCATCCAATAGGGCAT | 171 |
| cxcl1 | Forward sequence (5'-3'): GCACCCAAACCGAAGTCAT  Reverse sequence (5'-3'): GTGTTGTCAGAAGCCAGCGT | 179 |
| cxcl2 | Forward sequence (5'-3'): CCCAGACAGAAGTCATAGCCA  Reverse sequence (5'-3'): CTCCTCCTTTCCAGGTCAGTTA | 131 |
| CD45 (Ptprc) | Forward sequence (5'-3'): ATCATCGCCAGCATCTATCC  Reverse sequence (5'-3'): CATCCTGCTTGCCTCCATC | 103 |
| TGFβ-R (Tgfbr2) | Forward sequence (5'-3'): AGGACGACCGCTCCGACA  Reverse sequence (5'-3'): GACAGCCACGGTCTCAAACT | 167 |
| RORα | Forward sequence (5'-3'): TGGCTTCAGGAAAAGGTAAAA  Reverse sequence (5'-3'): AGTCGCACAATGTCTGGGTATA | 200 |
| Eotaxin (Ccl11) | Forward sequence (5'-3'): GAATATCAGCACCAGTCGCC  Reverse sequence (5'-3'): CCTCAATAATCCCACATCTCCT | 164 |
| GAPDH | Forward sequence (5'-3'): AAGAAGGTGGTGAAGCAGG  Reverse sequence (5'-3'): GAAGGTGGAAGAGTGGGAGT | 111 |
